# Predicting Tissue-Specific mRNA and Protein Abundance in Maize: A Machine Learning Approach

**DOI:** 10.3389/frai.2022.830170

**Published:** 2022-05-26

**Authors:** Kyoung Tak Cho, Taner Z. Sen, Carson M. Andorf

**Affiliations:** ^1^Department of Computer Science, Iowa State University, Ames, IA, United States; ^2^USDA-ARS, Crop Improvement and Genetics Research Unit, Albany, CA, United States; ^3^USDA-ARS, Corn Insects and Crop Genetics Research Unit, Ames, IA, United States

**Keywords:** maize genetics, gene expression, protein abundance, mRNA abundance, machine learning

## Abstract

Machine learning and modeling approaches have been used to classify protein sequences for a broad set of tasks including predicting protein function, structure, expression, and localization. Some recent studies have successfully predicted whether a given gene is expressed as mRNA or even translated to proteins potentially, but given that not all genes are expressed in every condition and tissue, the challenge remains to predict condition-specific expression. To address this gap, we developed a machine learning approach to predict tissue-specific gene expression across 23 different tissues in maize, solely based on DNA promoter and protein sequences. For class labels, we defined high and low expression levels for mRNA and protein abundance and optimized classifiers by systematically exploring various methods and combinations of k-mer sequences in a two-phase approach. In the first phase, we developed Markov model classifiers for each tissue and built a feature vector based on the predictions. In the second phase, the feature vector was used as an input to a Bayesian network for final classification. Our results show that these methods can achieve high classification accuracy of up to 95% for predicting gene expression for individual tissues. By relying on sequence alone, our method works in settings where costly experimental data are unavailable and reveals useful insights into the functional, evolutionary, and regulatory characteristics of genes.

## 1. Introduction

In the era of big data, machine learning is increasingly becoming a valuable tool to model biology and make powerful data-driven predictions, especially in genomics. Recent work has provided insight and understanding on a wide range of biological problems (Yip et al., 2013; Bastanlar and Ozuysal, 2014; Libbrecht and Noble, 2015; Eraslan et al., 2019; Avsec et al., 2021). Some of these methods are sequence-driven while others use large sets of complementary data that rely on the machine learning or deep learning approaches to identify the relevant features. For example, some recent machine learning based approaches have been used for gene annotation and classification in maize, which is a top production grain crop globally and an organism of historical and cultural importance. The methods include a random forest classifier based on methylation and histone modification patterns (Sartor et al., 2019), a natural language processing method on cell regulatory functions (Mejía-Guerra and Buckler, 2019), and a deep learning method for gene expression prediction (Washburn et al., 2019). Sartor et al. (2019) proposed gene expression classifiers based on sequence and chromatin information including DNA methylation data, histone modifications, and transcription-factor binding sites. Mejía-Guerra and Buckler (2019) introduced a k-mer grammar analysis model, which uses “bag-of-k-mers” and “vector-k-mers” models to annotate regulatory regions in maize. The “bag-of-k-mers” method focuses on k-mer frequency while the “vector-k-mers” model considers the frequency of k-mer pairs (co-occurrence of k-mer pairs). Washburn et al. (2019) proposed “gene-family guided splitting” and “ortholog contrasts” approaches for mRNA expression prediction.

Although early work has shown promise, an overgrowing need still exists to identify, label, and classify the products of high throughput genome sequencing into biological knowledge in a fast and efficient way. There are two primary ways to measure gene expression: (1) mRNA abundance using RNA-seq and (2) protein abundance using mass spectrometry. Both methods provide functional clues and can be used to associate genes to functional characteristics, but the experimental determination of protein function and regulation significantly lags far from that of sequencing. This disparity is likely to continue for the foreseeable future. Hence, protein assignment to a biological functional label from sequences alone remains an important and challenging problem in functional genomics (Eisenberg et al., 2000; Hanson et al., 2009; Griesemer et al., 2018). Machine learning approaches have been successfully used to predict gene expression from sequence. To produce the best gene expression predictors, information across the broad gene regulatory structure is needed (Zrimec et al., 2020). Recent work has used both coding and non-coding sequences inclusing transcription factor (TF) binding sites (Holland et al., 2019), chromatin accessibility (Zhang et al., 2016), interactions across distal promotor sequences (Zrimec et al., 2020), and other cis-regulatory regions in promoter regions (de Boer et al., 2020). A challenge is to make the process quick, accurate, versatile, scalable, and updateable for annotations of genomes with limited experimental data. For this reason, new machine learning approaches to produce such functional assignments are still needed.

The *k*-mer approach has become a reliable way to represent biological sequence data for quick and accurate predictors across a broad set of classification tasks (Vinga and Almeida, 2003; Vervier et al., 2016; Shen et al., 2018; Wang et al., 2018; Mejía-Guerra and Buckler, 2019; Alam and Chowdhury, 2020). The approach strikes a balance between accurate, rapid, and easy-to-understand classification (Wang et al., 2020) and can be paired with other deep learning techniques to generate reliable classifiers (Shen et al., 2018). Although *k*-mer methods perform well on various classification tasks, the use of non-sequential data to build classification models increases prediction performance. For example, in two of the studies (Sartor et al., 2019; Washburn et al., 2019), epigenetics and regulatory information were used to predict expression. However, a more challenging problem is to create models that scale to large genomics data or less supported genomes that have minimal experimental data, such as open chromatin, DNA methylation, histone modification, and transcription-factor binding sites. Experimental data can be very costly to produce in both time (growing plants, collecting tissues, sequencing, analyzing, etc.) and cost. Scaling the research at a pan-genomic scale exacerbates the cost by working on larger sets of accessions. For this reason, we chose to focus on developing a sequence-only-based approach with relatively simple models (for example, Bayesian) that scale well and are constructed and tested quickly. Our sequence-only-based approach has the key advantages that it can work on any set of genome annotations regardless of supporting data such as epigenetics and regulatory information.

Our approach extends existing methods for predicting gene expression (de Jongh et al., 2020) in three significant ways. First, most methods treat “expression prediction” as a binary problem—classifying genes as expressed or not based on a single threshold value. This classification does not consider where or under which conditions a gene is expressed or its level of expression. Our method leverages the gene expression tissue atlas data (Walley et al., 2016) to determine how well tissue-specific mRNA and protein abundance can be predicted. Second, our method looks at various ranges of expression. We build different models based on 5% increments of the top and bottom expressed genes in each tissue (e.g., top 95, 90, 85%, etc.). Third, in addition to using 5' DNA promoter sequences, our methods explore *k*-mer frequencies of the translated protein sequences.

For this study, we defined the problem as building classification methods to predict tissue-specific mRNA and protein abundance based on sequential features. We set out to develop transcriptome-level classifiers that can quickly identify common features within genes that are predictive of the expression in specific tissues. For our training data, we used DNA promoter and protein (peptide) sequences from the maize B73 reference genome (Schnable et al., 2009; Tello-Ruiz et al., 2018). Class labels were defined by experimental mRNA and protein abundance levels from an expression atlas of 23 maize tissues (Walley et al., 2016). The labels were assigned to each gene separate and independent of each other. To optimize the classification models, a systematic approach was used to evaluate performance across multiple *k*-mer lengths, inputs (DNA promoter vs. protein sequence), class labels (mRNA vs. protein abundance), and expression thresholds. A final classifier was built using a two-phase approach by combining results from sub-classifiers using different parameter combinations with common machine learning methods (Quinlan, 1986; Cortes and Vapnik, 1995; Friedman et al., 1997; Mucherino et al., 2009; Smith and Frank, 2016) for each of the 23 tissues. The goal of this study is predicting tissue-specific gene expression. A gene can be expressed in multiple tissues, so we built classifiers using classifications based on 5% expression increments for both high and low expressed genes. These increments allow a user to choose between high-confidence predictions vs. high-coverage predictions. We demonstrate that our fast, highly scalable approach accurately predicted tissue-specific gene expression in maize. The performance of these experiments show promise of applying these methods to other plant and crop model species.

## 2. Materials and Methods

**Figure 1:**
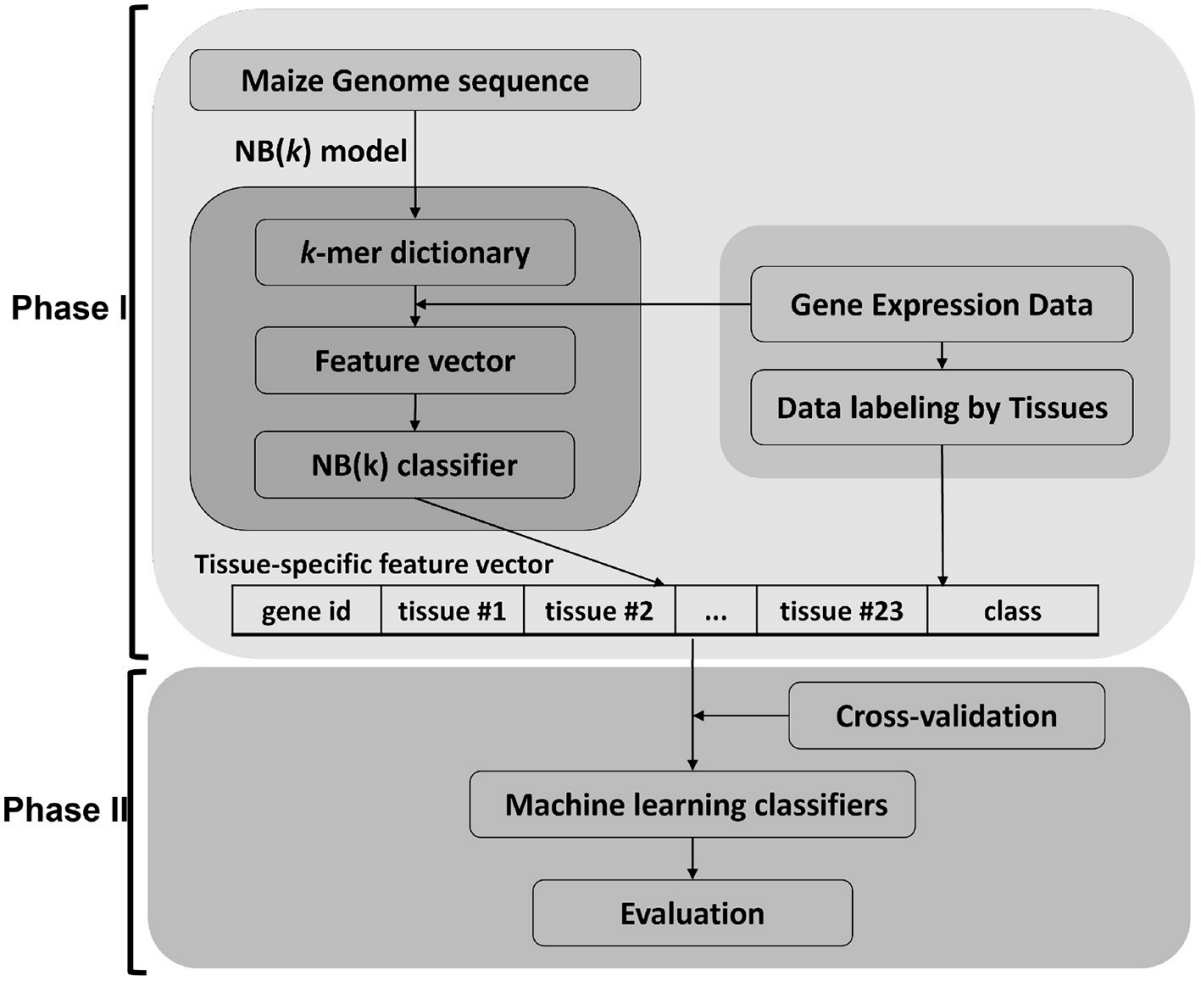
Framework of *t*NB(*k*) model. The approach for this paper is divided in two major parts. Phase I uses the NB(*k*) implementation of a *k* − 1 Markov model to build classifiers that predict tissue-specific gene expression of genes based on *k*-mer subsequences of either DNA or protein. The results from each tissue-specific classifier were used to generate a new feature vector that was in turn used as input to a Phase II classifier. For the Phase II classifier we showed results for 5 common machine learning approaches, but other machine learning approaches could be applied in the second phase.

This work focused on using maize as a model to develop sequence-only based approaches from 5' DNA promoter sequences and translated protein sequences. The data was downloaded through GenBank in FASTA format and re-structured into an internal database. The gene expression tissue atlas data (Walley et al., 2016) was used for data labeling for gene and protein expression. The experiments were designed by grouping genes into 12 different percentile groups based on (top six and bottom six by increments of 5%) mRNA abundance (RA) and protein abundance (PA) in 23 tissues. A gene is assigned to either the high expressed or low expressed class by labeling data per tissue. These class labels are used to build training and test data sets for machine learning (Phase I) using the NB(*k*) implementation of the *k* − 1 Markov model (Andorf et al., 2007, 2013). The binary outputs from these classifiers were used to form a feature-vector as input to a second stage (Phase II) machine learning classifier. In the Phase II method, four classical machine learning approaches (decision tree, Bayesian networks, k-nearest neighbor, and support vector machine) were evaluated (see Figure 1).

A challenge of evaluating a two-phase machine learning approach is balancing the tradeoff between overfitting and underfitting caused by using adequate data to build strong models, yet using proper data to evaluate the respective models. To use a standard set-aside validation framework for our methods would require using validation data for each fold in the cross-validation approach. This is needed because the second stage is dependent on the output of the first stage. For example, in a 10-fold cross-validation framework, 90% of the data is used for training and 10% for testing. To evaluate using a validation set, the first stage training would use the 10% second stage testing set to build the first phase models. Therefore to validate 1% of the data, only 9% of the total data would be used vs. the typical 90/10 ratio used in cross-validation. In this study, we focused on a systematic approach to evaluate across thousands of parameter combinations. Using only 9% of the data for first stage training was inadequate to train proper models, so we maintained distinct data representations for each stage to limit bias.

### 2.1. Data

To describe tissue-specific expression patterns, we considered both mRNA and protein abundance which were measured by RNA-seq data labeled with FPKM (Fragments per kilobase of transcript per million) and protein abundance data labeled with dNSAF (Distributed Normalized Spectral Abundance Factor) units, respectively, in 23 different tissues. The original expression data was from the gene expression tissue atlas data (Walley et al., 2016). The data was remapped to B73 RefGen v4 (Jiao et al., 2017) by (Walsh et al., 2020) and downloaded from MaizeGDB (Portwood et al., 2019). Each gene had 23 value pairs of tissue-specific RA and PA data. The RA data consisted of 39,324 genes and each gene had at least one FPKM value for any of the 23 tissues and the PA data had at least one dNSAF value for 14,815 genes.

Using the expressions (RA and PA) data, we built multiple models based on the top and bottom six percentiles of expression cutoffs at 5% increments (e.g., top 5% and/or bottom 10%). Table 1 shows the 95% cutoffs per tissue used in class labeling and the total number of genes with the tissue-specific measurement. See Supplementary Data 1 for full cutoffs information. A gene is labeled as an expressed (positive class) if the expression measure is greater than or equal to the cutoffs. Otherwise it is assigned into an unexpressed class (negative class). Each tissue has its own set of cutoff values per expression type (RA or PA). Using the expression cutoff setup makes an unequal number of positive and negative samples. Such an unbalanced data would causes bias in our experiments. For this reason, we constructed balanced datasets by randomly selecting an equal number of negative examples per tissue.

**Table 1:** A table describing the developmental atlas of maize data set.

**Tissue no**.	**Tissue name**	**Type**	**mRNA abundance**		**Protein abundance**	
			**Gene count**	**FPKM 95% cutoff**	**Gene count**	**dNSAF 95% cutoff**
1	6–7 internode	Internode	21,823	133.98	6,590	3,150.38
2	7–8 internode	Internode	21,738	137.49	7,241	3,035.73
3	B73 mature pollen	Pollen	9,075	231.50	4,346	4,440.14
4	Ear Primordium 2–4 mm	Ear	21,932	106.30	7,047	3,079,12
5	Ear Primordium 6–8 mm	Ear	22,472	101.76	8,129	2,577.80
6	Embryo 20 DAP	Ear	22,281	117.30	8,306	2,606.36
7	Embryo 2038 DAP	Ear	21,766	93.25	5,953	2,878.06
8	Endosperm 12 DAP	Ear	20,382	134.28	6,743	3,227.58
9	Endosperm crown 27 DAP	Ear	15,797	54.04	5,815	3,272.29
10	Female spikelet collected on day as silk	Ear	22,478	151.33	6,876	3,099.21
11	Germinatin Kernels 2 DAI	Ear	23,329	115.48	6,105	3,207.12
12	Leaf zone 1 (symmetrical)	Leaf	21,199	104.13	8,194	2,603.29
13	Leaf zone 2 (stomatal)	Leaf	21,369	99.13	8,879	2,517.95
14	Leaf zone 3 (growth)	Leaf	21,933	110.77	9,108	2,378.23
15	Mature leaf 8	Leaf	21,397	108.48	5,687	3,038.26
16	Pericarp/Aleurone 27 DAP	Ear	21,486	70.92	8,300	2,768.81
17	Primary root 5 days	Root	22,011	142.62	6,753	3,214.04
18	Root—cortex 5 days	Root	21,382	148.22	7,233	3,247.64
19	Root—elongation zone 5 days	Root	19,649	174.17	7,512	3,012.97
20	Root—Meristem zone 5 days	Root	20,009	145.86	7,575	3,164.69
21	Secondary root 7–8 days	Root	22,117	144.15	6,041	3,532.52
22	Silk	Silk	22,202	144.23	6,717	3,246.13
23	Vegetative Meristem 16–19 days	Meristem	20,963	131.48	7,305	3,032.82

*The data set is based on 23 tissues. We further grouped related tissues into 6 broad tissue types. The table shows the number of genes with either a FPKM or dNSAF for each tissue, these values correspond to mRNA or protein abundance values, respectively. The 95% columns show the cutoff value used to subdivide the data into the top 5% for each expression type.*

### 2.2. Data Preparation

The core input for the models are sequence-based *k*-mers. The frequency of *k*-mers in a given class was estimated by the observed counts of the training data set. Two sets of sequences were used. The first set was based on the translated protein sequences. The *k*-mer frequencies were based on the counts of amino acid, dimer, trimer, tetramer, etc. (*k* = 1, 2, 3, 4, etc., respectively). The second set was the 5' DNA promoter sequences from the 5kb region upstream of a given gene model's start site. The *k*-mer values ranging from 3 to 7 were tested. The *k*-mer frequencies for these two data sets were calculated for each of the 23 tissues and stored in a relational database. Supplementary Figure 1 shows the database schema of the key tables that store the sequence data and RA/PA labeling data.

We used a simple encoding of DNA promoter and protein sequences using the probability distribution of short (*k*-letter) subsequences (*k*-mers) of either nucleotides or amino acids. In our experiments, we reported results for *k*-values ranging from 3 to 7 for both protein and DNA promoter sequences. Larger values of *k* were not considered, because there was insufficient data to reliably estimate the model parameters. The number of all possible cases of *k*-mers for DNA sequence is 4^*k*^ because DNA has four bases, while there are 20 amino acids and a protein sequence can have up to 20^*k*^ *k*-mers. Based on classification performance, we identified the optimal window sizes of *k* = 3 for protein sequence and *k* = 3, …, 7 for the DNA promoter sequences.

### 2.3. Phase I: *k* − 1 Markov Model

We propose *k* − 1 Markov model to extract features from maize gene sequence and apply it to Naive Bayes model with *k*-mer frequency data called *k*-mer Naive Bayes model, NB(*k*). The simplest way to apply Naive Bayes classifier for gene sequence data is to consider a single alphabet. Equation (1) shows classifier for a given sequence *S* = *s*_1_, …, *s*_*n*_ with class *c*_*j*_ ∈ *C*.


(1) $$\begin{array}{l} c_{NB}\left(S\right)=\arg\underset{c_{j}\in C}{\max}P\left(c_{j}\right)\overset{n}{\underset{i=1}{\prod }}P_{\alpha }\left(S_{i}=s_{i}|c_{j}\right) \end{array}$$

where *C* = {*c*_0_, *c*_1_} which correspond to unexpressed and expressed, respectively. *c*_*NB*_ classifier considers only one alphabet to compute probability, and we extended this with the *k*-mer model which covered frequencies based on *k*-mers. For advanced feature extraction from gene sequence (protein and promoter), we first computed the *k*-mer frequency for all *k*-mers for given sequence *S* and applied Naive Bayes classifier with class *c*_0_ and *c*_1_. However, consecutive *k*-mers share (*k* − 1) letters with each and therefore are not independent of each other. Here, we used the NB(*k*) classifier which reduces possible bias by dividing those shared parts (*k* − 1-mers) as follows:


(2) $$\begin{array}{l} c_{NB\left(k\right)}\left(\bar{S}\right)\\ =\arg\underset{c_{j}\in C}{\max}P\left(c_{j}\right)\frac{\prod_{i=1}^{n-k+1}P_{\alpha }\left(S_{i}=s_{i},\ldots ,S_{i+k-1}=s_{i+k-1}|c_{j,k}\right)}{\prod_{i=2}^{n-k+1}P_{\alpha }\left(S_{i}=s_{i},\ldots ,S_{i+k-2}=s_{i+k-2}|c_{j,k-1}\right)} \end{array}$$

and Supplementary Figure 2 shows the mechanism of the NB(*k*) classifier.

### 2.4. Phase II: *t*NB(*k*): Two-Phase NB(*k*) Classifier

The Phase I model uses single tissue data per prediction. We wanted to extend this approach to consider other tissue data. The two-phase approach combines predictions across tissues, taking advantage of potential expression patterns. We constructed a two-phase NB(*k*) approach called *t*NB(*k*). First, we built a tissue-specific feature vector for each gene in the first phase using the NB(*k*) classifier. The feature vector consisted of 25 elements corresponding to gene id, predicted gene expressions by NB(*k*) classifier for 23 tissues, and assigned tissue. In the second phase, the class labels are the same as the first phase (binary labels specific to each tissue, *C* = {*c*_0_, *c*_1_}), and we performed experiments in Weka 3.8 with several classifiers (Bayesian networks, support vector machine, decision tree, and k-nearest neighbor classifier).

The gene expression and protein abundance datasets had at least one measured value of tissue for each gene, but not all genes had 23 measured values corresponding to 23 tissues. Some of the tissue-specific vectors had missing values, and we used mean imputation for missing values. See Supplementary Datas 2, 3 for the performance comparison of imputation vs. without imputation.

## 3. Results and Discussion

To discover the relationship between sequence and tissue-specific expression, we designed an approach based on the observed frequencies over a range of DNA or protein *k*-mer sequences in classes defined by mRNA and protein abundance levels (see Section 2.1). We tested this approach with different features: input type (promoter and protein sequences), tissue type, expression level (upper or lower), *k*-mer size, and machine learning algorithms. With these factors, we established eight experiment groups. Each group has genes that are expressed in the same tissue at similar levels. Table 2 summarizes the experimental setup for each group. We used two classification models: Phase I uses a simple Markov model for initial classification, and Phase II builds classifiers trained on Phase I predictions (see Section 2). Each phase has four experiments based on the combination of using two different sequence representations (protein or DNA promoter) and two class labels (mRNA or protein abundance). The experiments build individual classifiers for the 23 maize tissues with various *k*-mer sizes, identifying the top and bottom 5–30% abundance for each class type. The Phase II method builds classifiers with four common machine learning techniques (decision trees, Bayesian networks, k-nearest neighbor, and support vector machines). In total, each Phase I experiment generated 5,520 classifiers, and the Phase II experiments created 22,080 classifiers. Our methodology used 10-fold cross-validation on balanced datasets derived from 39,324 maize genes, and were evaluated with standard metrics of accuracy, precision, recall, and F-measure. See Supplementary Data 4 for a complete set of results for all the experiments and classifiers.

**Table 2:** A table of the eight experiment types used in this paper.

**Method**	**Sequence representation**	**Class labels**	**Parameters**
Phase I	Protein sequence	mRNA abundance Protein abundance	*k* = 3, ..., 7 Expression cutoff: Top 5–30%; Bottom 5–30%
	DNA promoter	mRNA abundance Protein abundance	*k* = 3, ..., 7 Expression cutoff: Top 5–30%; Bottom 5–30%
Phase II	Protein sequence	mRNA abundance Protein abundance	*k* = 3, ..., 7 Expression cutoff: Top 5–30%; Bottom 5–30%
	DNA promoter	mRNA abundance Protein abundance	*k* = 3, ..., 7 Expression cutoff: Top 5–30%; Bottom 5–30%

*We use two methods (Phase I and II) to predict tissue-specific gene expression (mRNA and protein abundance) for genes using k-mer sequences (DNA promoter and protein). In total there are eight experiments based on the combinations of method (Phase I or II), input type (DNA promoter or protein sequence), and output class (mRNA or protein abundance). Each experiment uses a systematic approach for building classifiers with additional combinations of the following parameters: k-mer size ranging from 3 to 7 and 12 expression cutoffs based on 5% increments of the genes with the top and bottom expression values*.

### 3.1. *k*-mer Size Had an Effect on Performance

**Figure 2:**
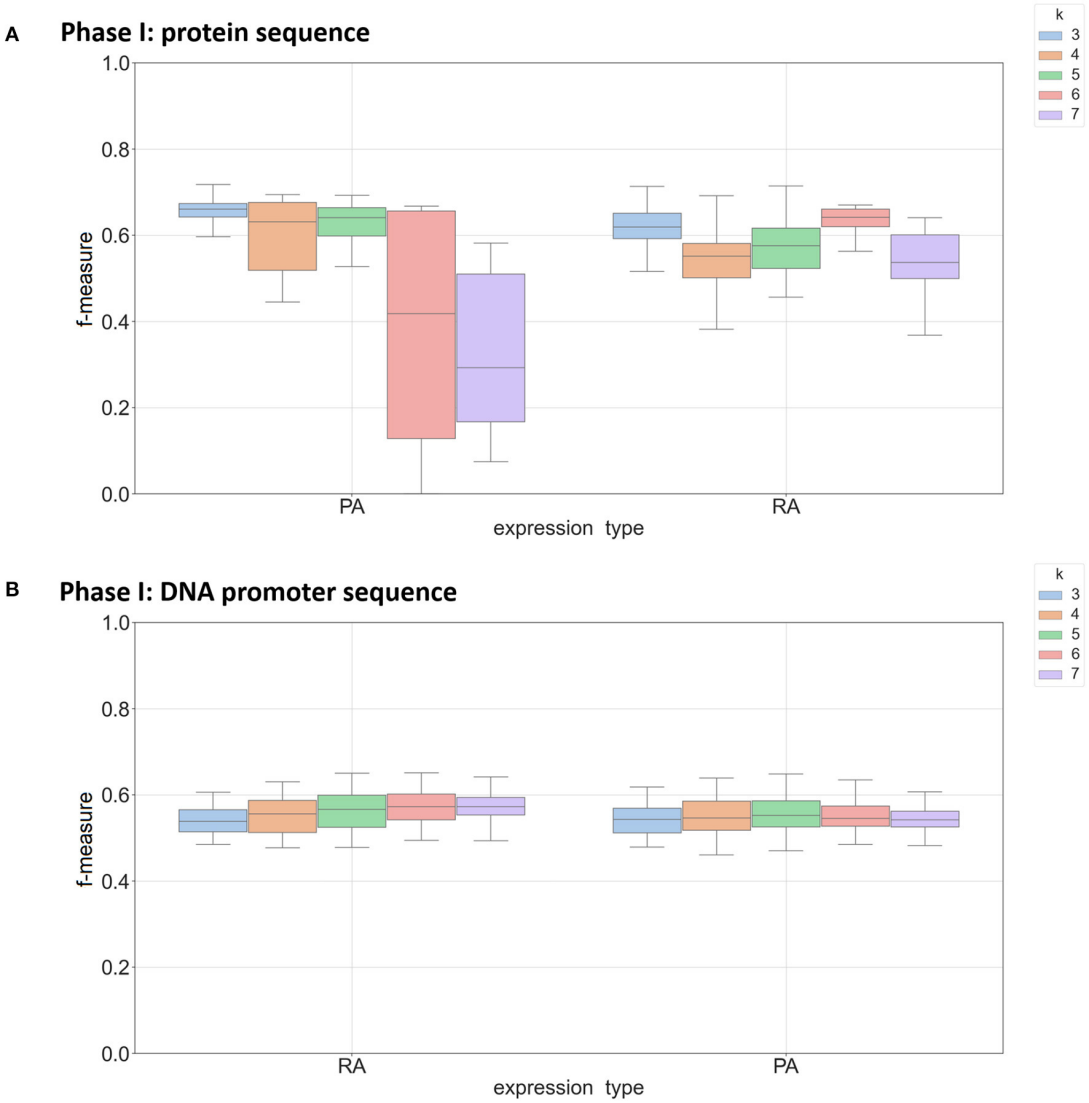
Box plot of F-measures for Phase I classifiers based on *k*-mer sizes of 3, 4, 5, 6, and 7. Each plot shows the interquartile range and mean of F-measures across all tissues using the top 5–30% and bottom 5–30% of expression cutoffs. Each graph is divided by classifiers predicting mRNA (RA) and protein (PA) abundance. Individual dots are outliers that are outside 1.5 times the interquartile range above the upper quartile or below the lower quartile. Panel **(A)** shows results using protein sequence as input and panel **(B)** show results using DNA promoter as input.

In the *k*-mer model, *k*-mer size is one of the most important parameters for feature extraction, and we focused on *k*-mer sizes ranging between 3 and 7. This range is consistent with other recent work using *k*-mers (Andorf et al., 2007). Figure 2 shows the range of F-measures for each value of *k* across the Phase I classifiers in predicting mRNA (RA) and protein abundance (PA). Figure 2A is based on classifiers using protein-based *k*-mer sequences and Figure 2B is based on DNA promoter-based *k*-mers. When *k* = 3 nucleotide sequences had the best performance for predicting both RA and PA with a sharp decrease in performance when *k* = 7. This result was expected as larger *k*-mer sizes create a full order (in terms of the alphabet size) greater features that need to be estimated, thus causing overfitting when not enough data is available. When using the DNA promoter sequence, the performance had less variance across *k*-mer sizes. The mean F-measure was between 0.55 and 0.60 for all *k*-mers in predicting both RA and PA with the best *k*-mer size was 6 for RA and 4 for PA. For the Phase II models, *k*-mer size had little effect on performance with little change in performance with the different *k*-mer sizes (F-measure near 0.85 across experiments). See Supplementary Figure 3 for complete results.

### 3.2. The Two-Phase Method Outperformed the One-Phase Approach

**Figure 3:**
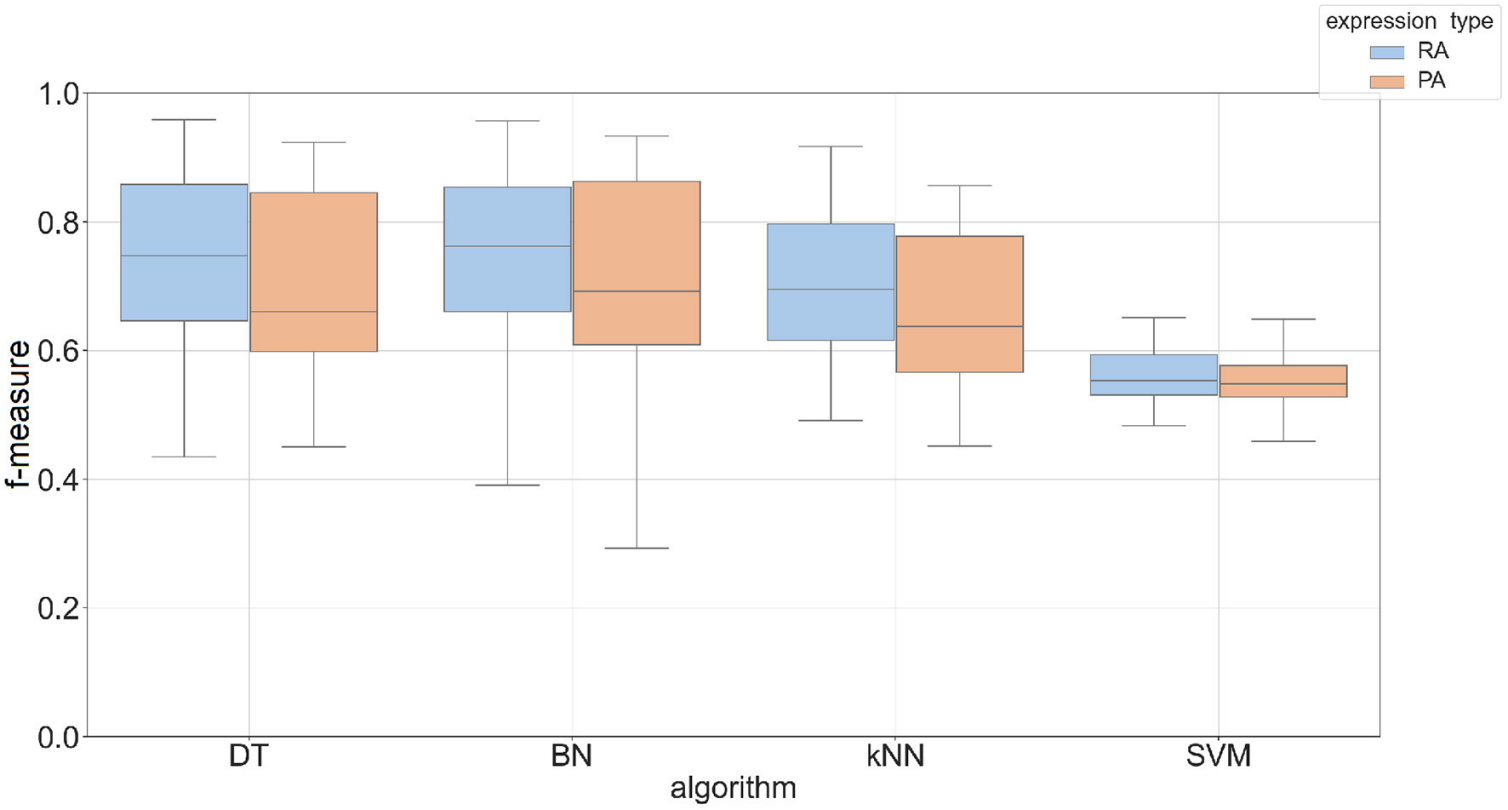
Box plot of F-measures for Phase II classifiers based on two expression types (RA, mRNA abundance; PA, protein abundance). The input type was DNA promoter sequence using a *k*-mer size of 5. Each plot shows the interquartile range and mean of F-measures across all tissues using the top 5–30% and bottom 5–30% of expression cutoffs. The x-axis is labeled by the implementation names of the four machine learning approaches (DT, Decision Tree; BN, Bayesian Network; kNN, k-Nearest Neighbors; SVM, Support Vector Machine).

The Phase I method created classifiers that predicted most tissues with an accuracy up to 60% with an F-measure between 0.5 and 0.7. The overall performance of predicting protein abundance is similar to mRNA abundance, but the protein abundance classifiers have a wider range of f-measures based on parameter choices (see Supplementary Figure 4). To take advantage of related tissues/conditions and genes co-expressed across different tissues (Li et al., 2016), we created a two-phase approach that uses the feature vector based on these 23 individual predictions. This feature vector was then used as input to an additional machine learning classifier (see Phase II method in Section 2). Four machine learning algorithms were compared in the Phase II method using Bayesian Network (BN), Decision Tree (DT), k-nearest neighbor (kNN), and support vector machine (SVM) classifiers. Figure 3 shows the performance by algorithm using promoter sequences for each expression type in the Phase II method. BN had the best performance across expression types using a *k*-mer size of 5 (mean F-measure of 0.75 for mRNA abundance and 0.73 for protein abundance) and SVM had the worst performance overall (mean F-measure of less than 0.57 for both expression types). We saw similar results when using protein sequence as input (see Supplementary Figure 5). Complete results for each of the machine learning approaches are provided in Supplementary Data 4. The results demonstrate that the BayesNet (BN) implementation had the best overall results. Therefore, we will present results from the BN method for the remaining sections when referring to Phase II results.

**Figure 4:**
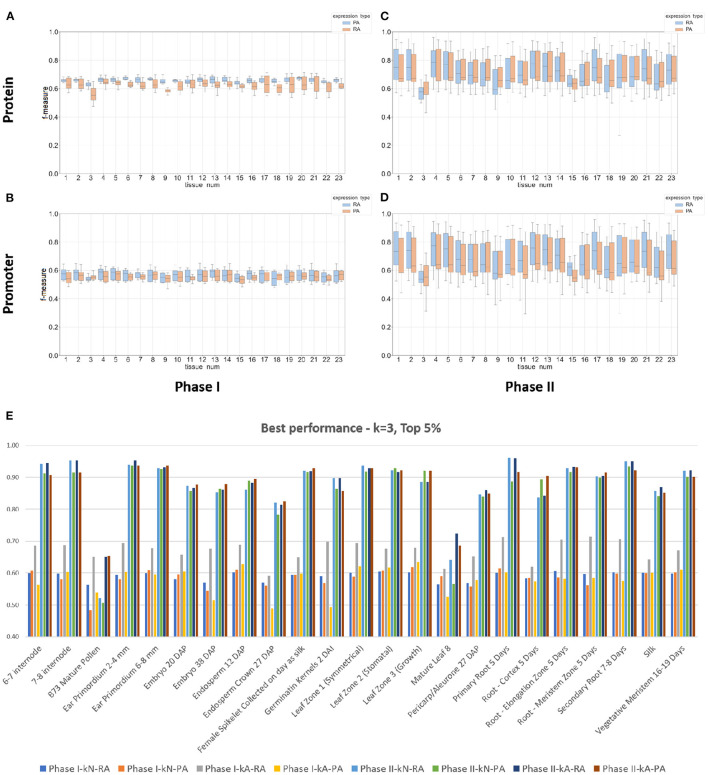
Box plots and a histogram of the best performing Phase I and II classifiers across all experiments. The Phase II method is based on Bayesian Network. Each plot shows the interquartile range and mean of F-measures across the top 5–30% and bottom 5–30% of expression cutoffs. The *k*-mer size used was 3 for protein sequence **(A,C)** and 3 for DNA promoter sequence **(B,D)**. The x-axis is labeled by tissue type (see Table 1 or Panel **(E)** for a full list of tissue names). Panels **(A,B)** are plots based on the Phase I method and Panels **(C,D)** are based on the Phase II method. Individual dots are outliers that are outside 1.5 times the interquartile range above the upper quartile or below the lower quartile. Panel **(E)** is a histogram of the best performing classifiers based on a fixed *k*-mer size of 3 and expression cutoff of Top 5%. The x-axis is labeled by tissue type. Each tissue displays the f-measure of the eight experiments based on the combinations of method (Phase I or II), input type (DNA promoter or protein sequence), and output class (mRNA or protein abundance).

From the data shown in Figure 4 and Table 3, the results from the Phase II method substantially outperformed the Phase I method in predicting either mRNA or protein abundance. Figures 4A–D display a side-by-side comparison of the Phase I and Phase II results across all experiments using the best *k*-value for each method. The figure shows the range of F-measures for the top 5%–top 30% expression values across each of the tissues for both mRNA (blue) and protein (orange) abundance. Figure 4E has a histogram view of the results from the best performing parameters (*k* = 3, Top 5% threshold, Bayesian Net). Table 3 is a tabular view of the accuracy and F-measure for both methods with the same fixed *k*-value (*k* = 3) and expression cutoff (5%). The histogram and table demonstrate that the Phase II methods outperformed Phase I regardless of input or class type on all tissues except pollen.

**Table 3:** Our methodology generated thousands of classifiers to predict gene expression for 23 tissues.

		**Phase I**								**Phase II**							
	**Input**	**Promoter**				**Amino acid**				**Promoter**				**Amino acid**			
	**Output**	**RA**		**PA**		**RA**		**PA**		**RA**		**PA**		**RA**		**PA**	
**Tissue #**		**FM**	**AC**	**FM**	**AC**	**FM**	**AC**	**FM**	**AC**	**FM**	**AC**	**FM**	**AC**	**FM**	**AC**	**FM**	**AC**
1	6-7 internode	0.60	0.57	0.61	0.58	0.69	0.74	0.56	0.68	0.94	0.94	0.91	0.92	0.95	0.95	0.91	0.91
2	7-8 internode	0.60	0.57	0.58	0.55	0.69	0.74	0.60	0.70	0.95	0.95	0.91	0.92	0.95	0.95	0.92	0.92
3	B73 Mature pollen	0.56	0.58	0.48	0.50	0.65	0.69	0.54	0.66	0.52	0.55	0.51	0.62	0.65	0.69	0.65	0.71
4	Ear Primordium 2–4 mm	0.59	0.57	0.58	0.55	0.69	0.73	0.60	0.70	0.94	0.94	0.94	0.94	0.95	0.95	0.94	0.94
5	Ear Primordium 6–8 mm	0.60	0.57	0.61	0.58	0.68	0.72	0.60	0.69	0.93	0.93	0.93	0.93	0.93	0.93	0.94	0.94
6	Embryo 20 DAP	0.58	0.56	0.60	0.57	0.66	0.72	0.61	0.70	0.87	0.88	0.86	0.87	0.87	0.87	0.88	0.88
7	Embryo 2038 DAP	0.57	0.57	0.54	0.56	0.68	0.72	0.52	0.66	0.85	0.86	0.86	0.87	0.86	0.87	0.88	0.88
8	Endosperm 12 DAP	0.60	0.59	0.61	0.58	0.69	0.74	0.63	0.72	0.86	0.87	0.89	0.89	0.88	0.89	0.89	0.90
9	Endosperm crown 27 DAP	0.57	0.56	0.56	0.54	0.59	0.66	0.49	0.64	0.82	0.83	0.78	0.80	0.81	0.83	0.82	0.84
10	Female spikelet collected on day as silk	0.59	0.57	0.59	0.57	0.65	0.72	0.60	0.70	0.92	0.92	0.92	0.92	0.92	0.92	0.93	0.93
11	Germinatin Kernels 2 DAI	0.59	0.56	0.57	0.56	0.70	0.74	0.49	0.65	0.90	0.90	0.86	0.87	0.90	0.90	0.86	0.86
12	Leaf zone 1 (symmetrical)	0.60	0.58	0.59	0.57	0.69	0.73	0.62	0.71	0.94	0.94	0.92	0.92	0.93	0.93	0.93	0.93
13	Leaf zone 2 (stomatal)	0.60	0.58	0.61	0.58	0.68	0.73	0.62	0.71	0.92	0.92	0.93	0.93	0.92	0.92	0.92	0.92
14	Leaf zone 3 (growth)	0.60	0.57	0.62	0.59	0.68	0.73	0.63	0.72	0.89	0.89	0.92	0.92	0.89	0.89	0.92	0.92
15	Mature leaf 8	0.56	0.55	0.59	0.57	0.61	0.67	0.53	0.67	0.64	0.70	0.57	0.65	0.72	0.74	0.69	0.74
16	Pericarp/aleurone 27 DAP	0.57	0.56	0.56	0.55	0.65	0.70	0.58	0.68	0.85	0.85	0.84	0.85	0.86	0.87	0.85	0.86
17	Primary root 5 days	0.60	0.57	0.61	0.59	0.71	0.76	0.60	0.71	0.96	0.96	0.89	0.89	0.96	0.96	0.92	0.92
18	Root—cortex 5 days	0.58	0.55	0.58	0.57	0.62	0.69	0.57	0.69	0.84	0.85	0.89	0.90	0.84	0.85	0.90	0.91
19	Root—elongation zone 5 days	0.61	0.58	0.59	0.56	0.70	0.76	0.58	0.69	0.93	0.93	0.92	0.92	0.93	0.93	0.93	0.93
20	Root—Meristem zone 5 days	0.60	0.57	0.56	0.53	0.71	0.76	0.58	0.69	0.90	0.91	0.90	0.90	0.90	0.91	0.92	0.92
21	Secondary root 7–8 days	0.60	0.57	0.60	0.57	0.71	0.75	0.58	0.69	0.95	0.95	0.93	0.94	0.95	0.95	0.92	0.92
22	Silk	0.60	0.57	0.60	0.57	0.64	0.71	0.60	0.70	0.86	0.87	0.84	0.85	0.87	0.88	0.85	0.86
23	Vegetative Meristem 16–19 days	0.60	0.57	0.60	0.58	0.67	0.73	0.61	0.71	0.92	0.92	0.90	0.91	0.92	0.92	0.90	0.91

*The table shows the results of the best set of classifiers which was achieved using a fixed k-mer size of 3 and expression cutoff of Top 5%. Each tissue displays the F-measure (FM) and accuracy (AC) of the eight experiments based on the combinations of method (Phase I or II), input type (DNA promoter or amino acid sequence), and output class (mRNA (RA) or protein abundance (PA)*.

The top two panels of Figure 4 show a range of F-measures when using an input based on protein sequence (Figures 4A,C) and the bottom two panels show performance based on DNA promoter sequence (Figures 4B,D). When using protein sequence as input, the Phase II approach had an average improvement in accuracy of 16.8% (RA) and 20.0% (PA) and F-measure improvement of 0.21 (RA) and 0.30 (PA). The Phase II method showed similar improvements when using promoter sequence as input. The average increase in accuracy was 31.2% (RA) and 31.1% (PA) and increased the F-measure by 0.28 (RA) and 0.28 (PA). The only tissue where Phase II performed worse than Phase I was pollen. The prediction performance for Phase II was superior to the Phase I method in classifying gene expression, and therefore, we will present the rest of the results with a focus on Phase II results using the Bayesian Net method.

### 3.3. Classifiers Trained From Protein Sequences Outperformed Similar Classifiers Based on Promoter Data

Our methods use training data from both DNA promoters and translated protein sequences. Previous studies (Meyer et al., 2013; Huminiecki and Horbanczuk, 2017; Mejía-Guerra and Buckler, 2019; N'Diaye et al., 2020; Schmidt et al., 2020; Avsec et al., 2021) have focused on sequences from promoter regions which are rich in regulatory regions associated with both gene enhancing and silencing. These regions are important in determining if and at what level a gene will be expressed. We built classifiers that predicted tissue-specific gene expression and therefore wanted to determine if protein subsequences could differentiate expression patterns across tissues. The results of our study show that tissue-specific predictions based on protein sequences consistently outperformed promoter-based predictions across a broad set of different parameters. For example, Table 3 shows performance of classifying genes with top 5% gene expression. The Phase I results show that classifiers using protein sequences as input had the best performance every time when predicting mRNA abundance and 61% of the time for protein abundance. For Phase II, protein sequence based classifiers had the best performance on 91% of the data for both mRNA and protein abundance data.

### 3.4. Classifiers Predicted Protein Abundance Better Than mRNA Abundance

Gene expression is the main driver for cellular functions. Many studies use RNA-seq data to measure protein abundance in a tissue or under a certain condition. The original study (Walley et al., 2016) from the maize expression atlas reported that mRNA abundance does not always tightly correlate with protein abundance. This result shows the importance of separately predicting mRNA and protein levels to understand gene function. Our results demonstrate that machine learning classifiers can be used to predict both mRNA and protein abundance across 23 different tissues. Figure 4 presents our best results in predicting both types of expression. When using promoter sequence as the input (Figure 4B), our methods performed slightly better in predicting protein abundance in 15 out of the 23 tissues. The sets of tissues with better performance in predicting mRNA abundance were intermode, ear, kernel, silk, and three out of the six root tissues. When using protein sequence as input (Figure 4D), the results were very similar: 16 out of the 23 tissues had a higher F-measure for protein abundance.

### 3.5. Classifiers Performed Better at Predicting High Expression Classes vs. Low Expression

**Figure 5:**
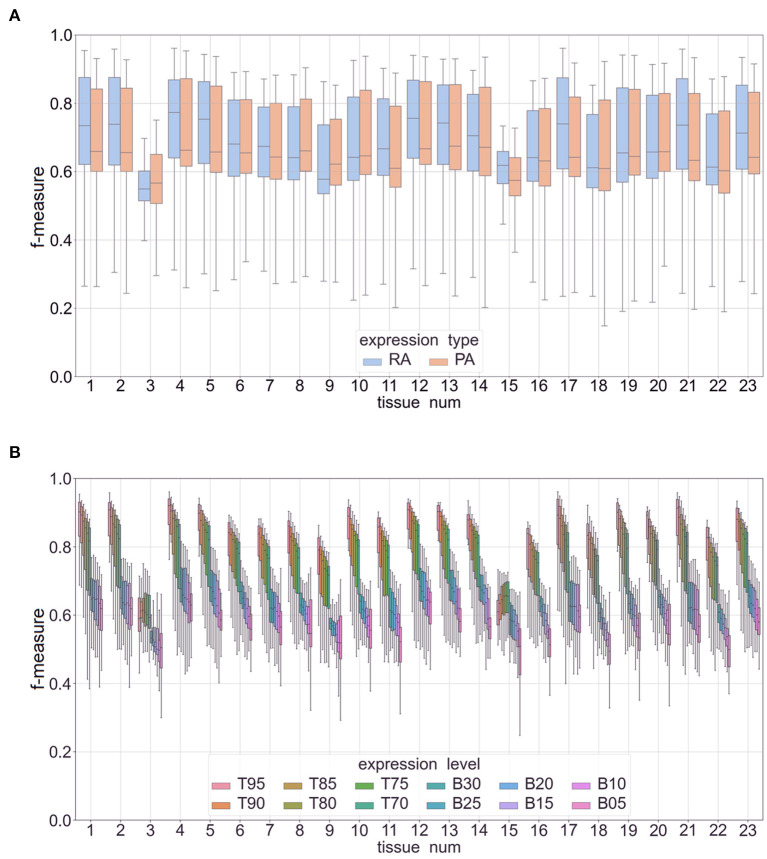
Box plots of F-measures for Phase II classifiers based on two expression types (RA, mRNA abundance; PA, protein abundance). The x-axis is labeled by tissue type (see Table 1 for a full list of tissue names). Panel **(A)** is a plot of interquartile range and mean of F-measures across the top 5–30% of expression cutoffs, bottom 5–30% of expression cutoffs, and *k*-mer sizes of 3–7. Panel **(B)** is a similar plot with separate box plots for each expression cutoff (top expression: T95–T70 and low expression: B30–B05).

Figure 5 shows the overall performance of our methods (Figure 5A) and performance broken down across the different expression cutoffs (Figure 5B). Although performance decreased as percentile value increased, our methods did well in predicting genes with a high expression (i.e., between 70% and 95%, denoted as T70 and T95, respectively. The models had a mean accuracy of 87.8% and F-measure of 0.89 in predicting the top 5% in mRNA abundance. In the top 5% of protein abundance prediction, the mean accuracy is 87.1% and F-measure is 0.88. The performance metrics decreased when predicting low expression (B30–B05, B denotes “bottom”). The mean accuracy was 65.0% with a F-measure of 0.67 for predicting low expressing genes. Supplementary Data 4 has the complete results including results on classifying combined features (e.g., high mRNA abundance with low protein abundance) and for each cutoff value. Supplementary Figure 4A shows a box-plot of the F-measure in predicting mRNA and protein abundance in Phase I. Supplementary Figure 4B shows the performance of different cutoffs: Top 5–30% (T95–T70) and Bottom 5–30% (B05–B30). The figure shows a clear decline in performance as the percentage increases. We hypothesize that there could be a tissue-specific signal in the DNA promoter and protein sequences that the machine learning classifiers can detect, but this signal is not as strong or that the variance of expression levels might be better explained by enhancers or other regulatory regions that we are not including in our analysis. Also, the combined feature classifiers did not perform as well as the high expression classifiers. We hypothesize that this is mainly due to the small and unbalanced nature of these subsets of data. For example, there are very few cases of low mRNA abundance with high protein expression.

### 3.6. The Two-Phase Method Could Reliably Identify Genes With High Expression in Most Tissues

The main aim of this study was to evaluate the effectiveness of sequence-based machine learning approaches to identify high/low transcript or protein levels across a wide range of tissues in maize. We were able to achieve this aim and identify the best features and parameters to construct tissue-specific classifiers. The best performing classifiers used a *k*-mer size of 3 for both protein sequences and DNA promoter sequences, expression cutoff of top 5%, and a two-phase approach using Baysian networks (see Table 3). Although the overall performance was good, some tissue-specific classifiers performed better than others. The classifiers based on internode data (tissues #1 and #2) had F-measures of 0.926 and 0.934, respectively. The root (tissues 17–21) classifiers had F-measures ranging from 0.869 and 0.939. Ear, embryo, and endosperm classifiers ranged from 0.848–0.941. Leaf classifiers also had good performance of 0.903–0.927 with the exception of tissue #15 (mature leaf 8). The three classifiers with the worst performance were based on pollen (tissue #3), mature leaf 8 (tissue #15), and endosperm crown 27 days after pollination (tissue #9) with F-measures of 0.582, 0.654, and 0.810, respectively. See Table 1 for the information of tissue type categories. Each of these three tissues are based on mature samples. It is worth noting that the pollen classifiers consistently performed significantly worse than any other tissue across all experiments. Other studies including (Walsh et al., 2020) have shown that as compared to other tissues, maize pollen has fewer expressed genes, a higher rate of expressed transcription factors, and that pollen mRNA abundance has low correlation with protein abundance. The same study showed that out of the same 23 tissues only “pollen” and “endosperm crown 27 days after pollination” had higher expression in the non-dominant subgenome of maize.

## 4. Conclusion

As sequencing technologies become increasingly affordable and assembly and annotation methods mature, more whole-genome assemblies and predicted gene sequences become available. Identifying which genes/proteins are expressed and under what conditions they are expressed is an important step to understand the functional and regulatory roles of the underlying genes and their impact on traits and phenotypes.

Six major findings emerged from our study: (1) *k*-mer size had an effect on performance; (2) the two-phase method outperformed the one-phase approach; (3) classifiers trained from protein sequences outperformed similar classifiers based on promoter data; (4) classifiers predicted protein abundance better than mRNA abundance; (5) classifiers substantially performed better at predicting high expression classes vs. low expression; and (6) our two-phase approach could reliably identify genes with high expression in most tissues.

Our machine-learning approach demonstrated that it is possible to reliably classify tissue-specific expression in maize. Where other prediction methods use experimental data, our computationally inexpensive approach is sequence-based and therefore can be used even when experimental data is unavailable. Our method performed well on predicting high-expression at a tissue level (top 5% of genes), and declined as we broadened the cutoff value of an expressed gene. These results suggest that high-expressed tissue-specific signals are in the DNA promoter sequence and/or the protein sequence), but the variance of expression within that tissue might have a weaker signal or be better explained by changes in other regulatory regions or mechanisms. Additionally, our systematic approach showed the roles of both input features (sequence type, sub-sequence size) and output features (expression type, expression cutoffs) in predicting gene expression. Our approach has strong potential to provide important insights into plant genetics, evolution, development and therefore expedite crop improvement and improve human health.

## Data Availability Statement

Publicly available datasets were analyzed in this study. This data can be found here: Genbank Bioproject PRJNA217053.

## Author Contributions

KC and CA were involved in project design. TS contributed to the interpretation of the data and results. KC performed the data processing, algorithm development, coding, testing, and analysis. CA provided the project administration and funding acquisition. All authors were involved in writing, reviewing, and editing of the manuscript. All authors contributed to the article and approved the submitted version.

## Funding

This research was supported by the US. Department of Agriculture, Agricultural Research Service, Project Numbers [5030-21000-068-00-D] through the Corn Insects and Crop Genetics Research Unit and [2030-21000-024-00-D] through the Crop Improvement and Genetics Research Unit. Mention of trade names or commercial products in this publication is solely for the purpose of providing specific information and does not imply recommendation or endorsement by the U.S. Department of Agriculture. USDA is an equal opportunity provider and Employer.

## Conflict of Interest

The authors declare that the research was conducted in the absence of any commercial or financial relationships that could be construed as a potential conflict of interest.

## Publisher's Note

All claims expressed in this article are solely those of the authors and do not necessarily represent those of their affiliated organizations, or those of the publisher, the editors and the reviewers. Any product that may be evaluated in this article, or claim that may be made by its manufacturer, is not guaranteed or endorsed by the publisher.

## Supplementary Material

Supplementary data 1 Full cutoffs information. Table 1.XLSX has full cutoffs information used in this study for mRNA (RA) and protein abundance (PA) data.

Supplementary data 2 The performance comparison of imputation for all expression levels. Table 2.XLSX has the performance comparison (FM: f-measure and AC: accuracy) of imputations versus without imputation. It is based on all expression levels, k-mer sizes of 3,4,5,6, and 7, and algorithms (BN: Bayesian Network; DT: Decision Tree; kNN: k-Nearest Neighbors; SVM: Support Vector Machine) for mRNA, protein abundance, and combined of both abundances.

Supplementary data 3 The performance comparison of imputation for Top 5%. Table 3.XLSX has the performance comparison (FM: f-measure and AC: accuracy) of imputations versus without imputation for only Top 5% expression level. It is based on k-mer sizes 3,4,5,6, and 7, and algorithms (BN: Bayesian Network; DT: Decision Tree; kNN: k-Nearest Neighbors; SVM: Support Vector Machine) for mRNA abundance and protein abundance.

Supplementary data 4 A complete set of results. Table 4.XLSX has a complete set of results for all the experiments and classifiers.

Supplementary Figure S1 Database schema of the key tables. Data Sheet 1.PDF shows the database schema of the key tables.

Supplementary Figure S2 Mechanism of k-1 Markov model (NB(k)). Data Sheet 1.PDF shows the mechanism of k-1 Markov model (NB(k)).

Supplementary Figure S3 Box plot of F-measures for Phase II based on k-mer sizes of 3,4,5,6, and 7. Image 1.PNG shows a box plot of F-measures for Phase II classifiers based on k-mer sizes of 3,4,5,6, and 7. Each plot shows the interquartile range and mean of F-measures across all tissues using the top 5–30% and bottom 5–30% of expression cutoffs. Each graph is divided by classifiers predicting mRNA (RA) and protein (PA) abundance. Individual dots are outliers that are outside 1.5 times the interquartile range above the upper quartile or below the lower quartile. Panel A shows results using protein sequence as input and panel B show results using DNA promoter as input.

Supplementary Figure S4 Box plot of F-measures for Phase II based on expression types with protein sequence. Image 2.PNG shows a box plot of F-measures for Phase II classifiers based on two expression types (RA: mRNA abundance; PA: protein abundance). The input type was protein sequence using a k-mer size of 3. Each plot shows the interquartile range and mean of F-measures across all tissues using the top 5–30% and bottom 5–30% of expression cutoffs. The x-axis is labelled by the implementation names of the four machine learning approaches (DT: Decision Tree; BN: Bayesian Network; kNN: k-Nearest Neighbors; SVM: Support Vector Machine).

Supplementary Figure S5 Box plot of F-measures for Phase I classifiers based on two expression types. Image 3.PNG shows a box plot of F-measures for Phase II classifiers based on two expression types (RA: mRNA abundance; PA: protein abundance). Each plot shows the interquartile range and mean of F-measures across all tissues using the top 5–30% and bottom 5–30% of expression cutoffs. The x-axis is labeled by the implementation names of the four machine learning approaches (DT, Decision Tree; BN, Bayesian Network; kNN, k-Nearest Neighbors; SVM, Support Vector).
