# Supplementary figures and images for: Predicting Tissue-Specific mRNA and Protein Abundance in Maize: A Machine Learning Approach
Source: Front Artif Intell. 2022 May 26;5:830170. doi: 10.3389/frai.2022.830170 (PMC9204276; doi:10.3389/frai.2022.830170)

## Database schema of the key tables

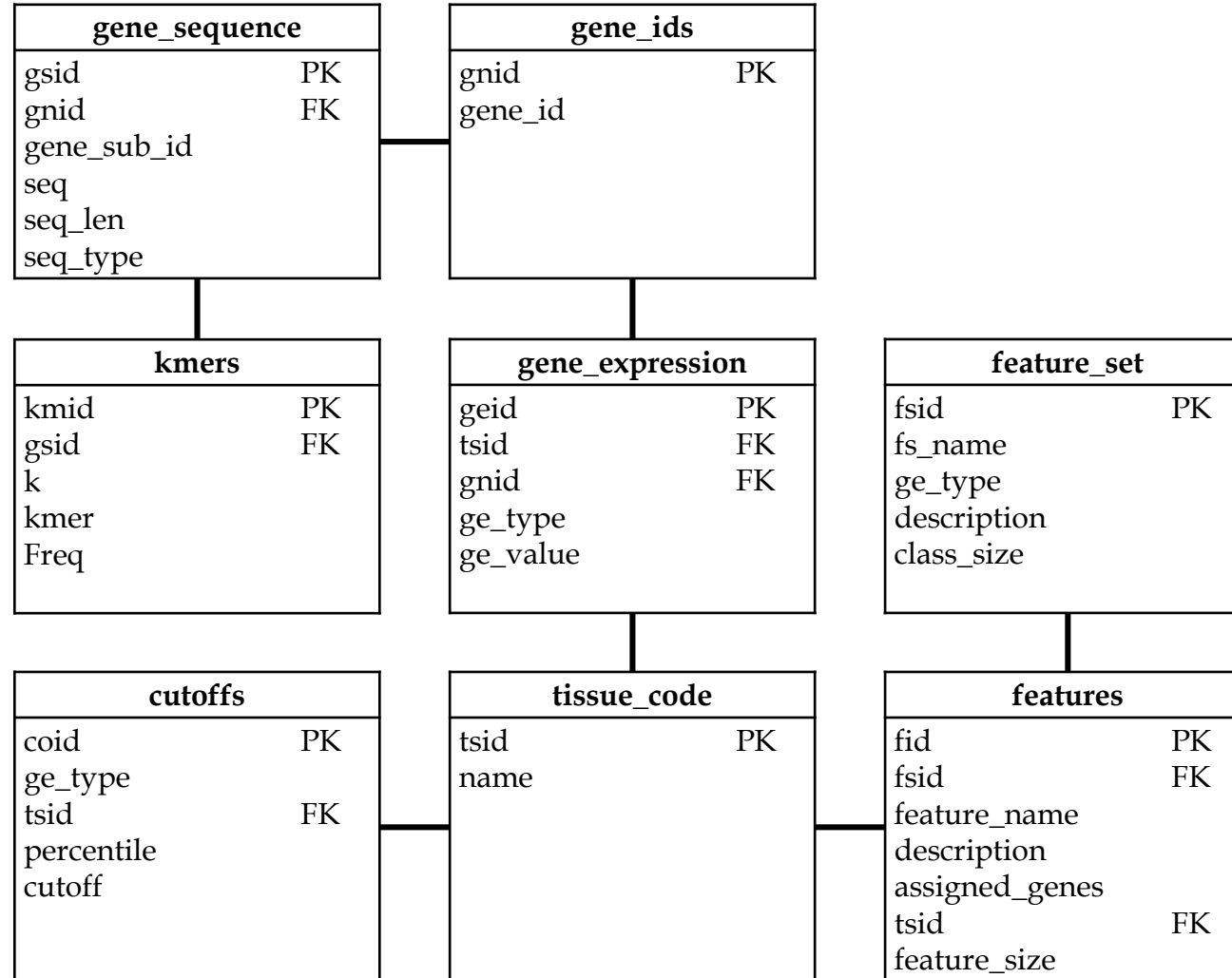

Supplement: Supplementary Figure S1 — Database schema of the key tables. Data Sheet 1.PDF shows the database schema of the key tables. [file Data_Sheet_1.PDF]

## Mechanism of k-1 Markov model (NB(k))

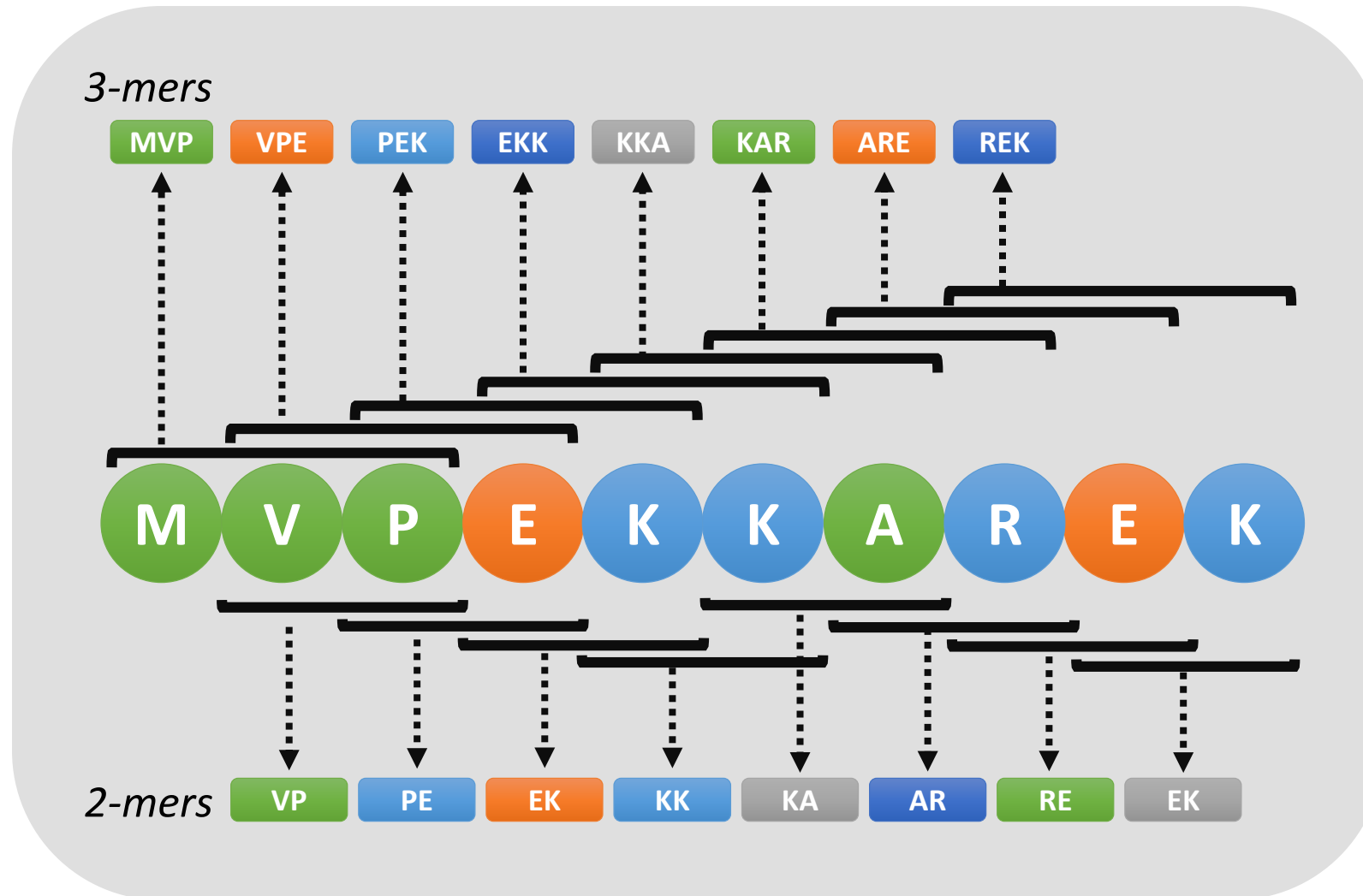

Supplement: Supplementary Figure S2 — Mechanism of k-1 Markov model (NB(k)). Data Sheet 1.PDF shows the mechanism of k-1 Markov model (NB(k)). [file Data_Sheet_2.PDF]

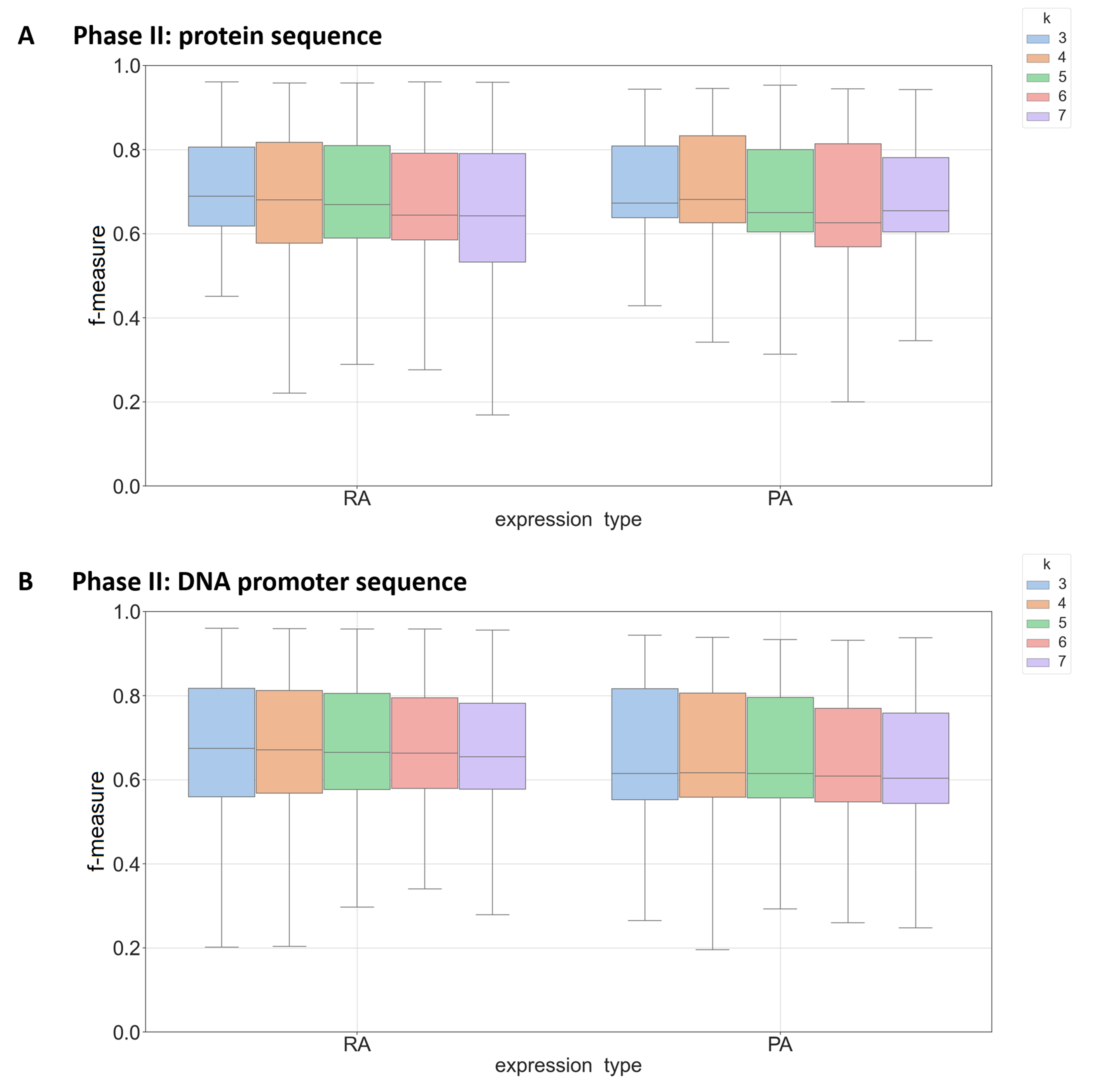

Supplement: Supplementary Figure S3 — Box plot of F-measures for Phase II based on k-mer sizes of 3,4,5,6, and 7. Image 1.PNG shows a box plot of F-measures for Phase II classifiers based on k-mer sizes of 3,4,5,6, and 7. Each plot shows the interquartile range and mean of F-measures across all tissues using the top 5–30% and bottom 5–30% of expression cutoffs. Each graph is divided by classifiers predicting mRNA (RA) and protein (PA) abundance. Individual dots are outliers that are outside 1.5 times the interquartile range above the upper quartile or below the lower quartile. Panel A shows results using protein sequence as input and panel B show results using DNA promoter as input. [file Image_1.PNG]

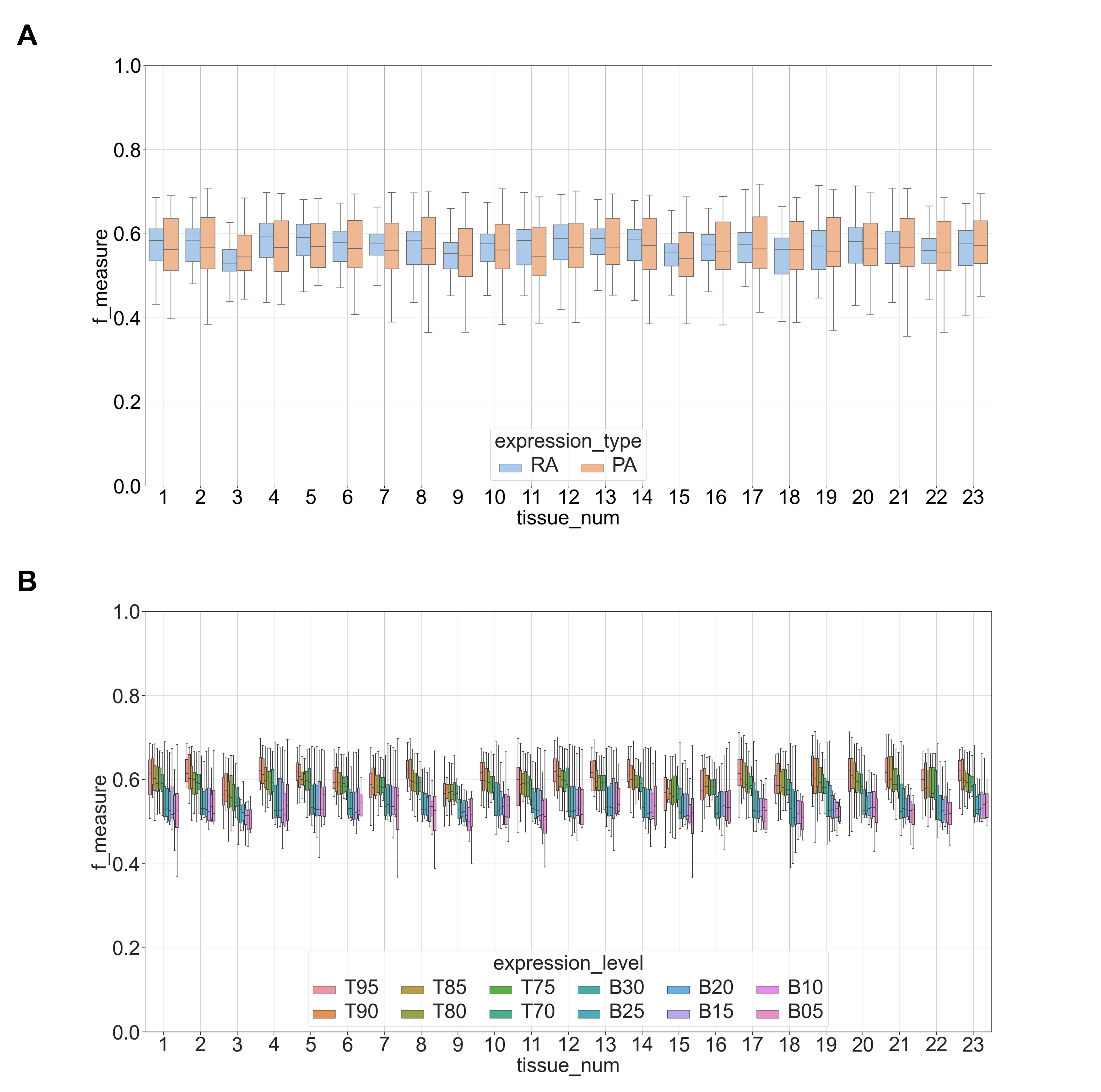

Supplement: Supplementary Figure S4 — Box plot of F-measures for Phase II based on expression types with protein sequence. Image 2.PNG shows a box plot of F-measures for Phase II classifiers based on two expression types (RA: mRNA abundance; PA: protein abundance). The input type was protein sequence using a k-mer size of 3. Each plot shows the interquartile range and mean of F-measures across all tissues using the top 5–30% and bottom 5–30% of expression cutoffs. The x-axis is labelled by the implementation names of the four machine learning approaches (DT: Decision Tree; BN: Bayesian Network; kNN: k-Nearest Neighbors; SVM: Support Vector Machine). [file Image_2.PNG]

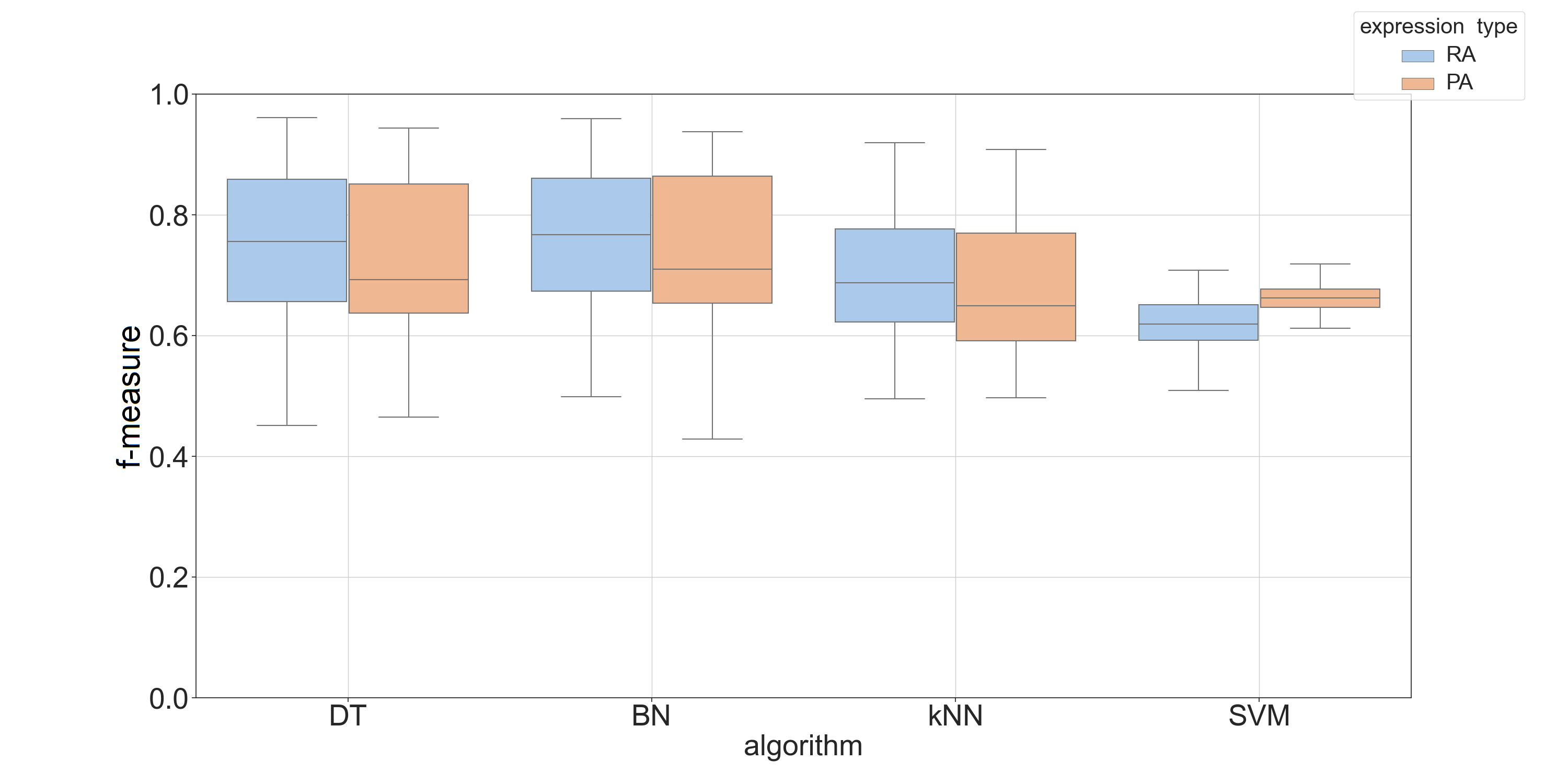

Supplement: Supplementary Figure S5 — Box plot of F-measures for Phase I classifiers based on two expression types. Image 3.PNG shows a box plot of F-measures for Phase II classifiers based on two expression types (RA: mRNA abundance; PA: protein abundance). Each plot shows the interquartile range and mean of F-measures across all tissues using the top 5–30% and bottom 5–30% of expression cutoffs. The x-axis is labeled by the implementation names of the four machine learning approaches (DT, Decision Tree; BN, Bayesian Network; kNN, k-Nearest Neighbors; SVM, Support Vector). [file Image_3.PNG]
